# Supplementary material for: Complementation of an Eisosomal Yeast pil1 Mutant and Characteristics of Eisosomal Distribution in Hyphae of Neurospora crassa Germinating from Two Different Spore Types
Source: J Fungi (Basel). 2023 Jan 22;9(2):147. doi: 10.3390/jof9020147 (PMC9964885; doi:10.3390/jof9020147)
Supplement: Supplementary file 1 [file jof-09-00147-s001.zip › jof-1918986-supplementary.pdf]

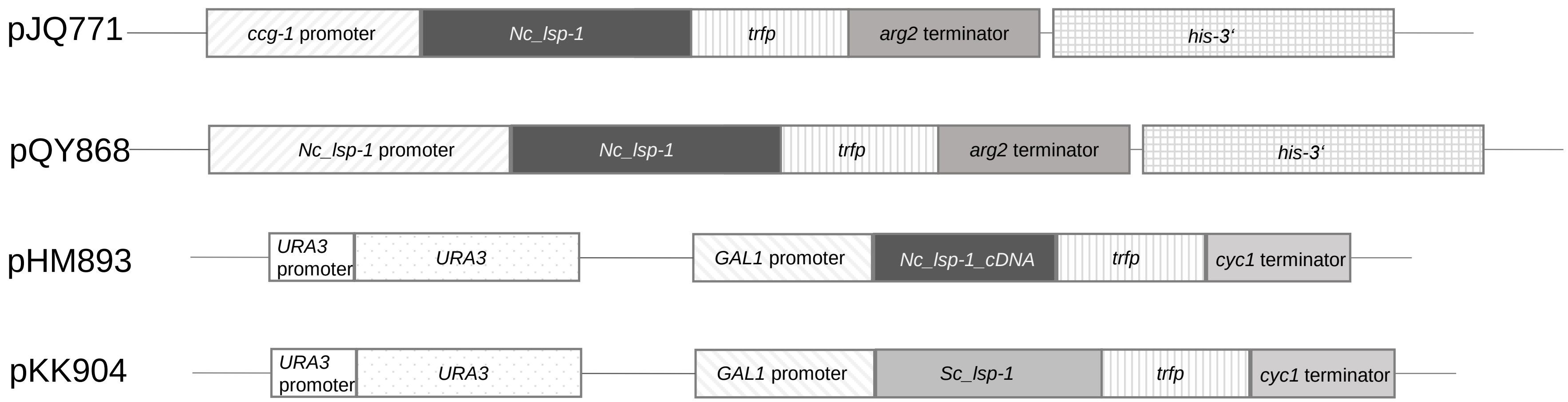

Supplementary Figure S1. Schematic representation of vectors created in the study. Vector components are indicated within the drawings. For selection in *N. crassa his-3* gene fragment was used. URA3 was used as selection marker in yeast. All vectors carry ampicillin resistency gene *bla* for selection. in *E*. *coli* (not indicated in the figure).

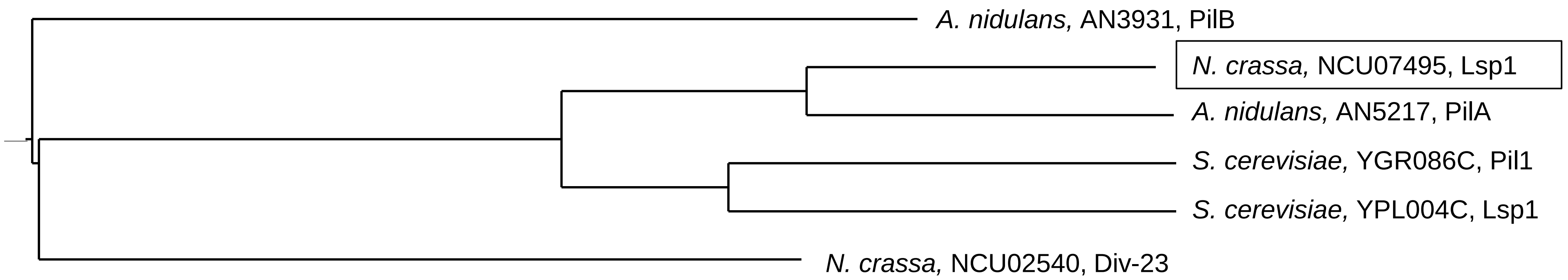

Supplementary Figure S2. Phylogenetic analysis results. LSP-1 protein in *N. crassa* is a homologue of the A. nidulans PilA, which is a core component of eisosomes. Both A. nidulans PilA and *N. crassa* LSP-1 are homologous to the yeast core eisosomal proteins Pil1 and Lsp1. Analized with Clone Manager, Multiway Alignment, Scoring matrix: BLOSUM62, Similarity significance value set to 60%.

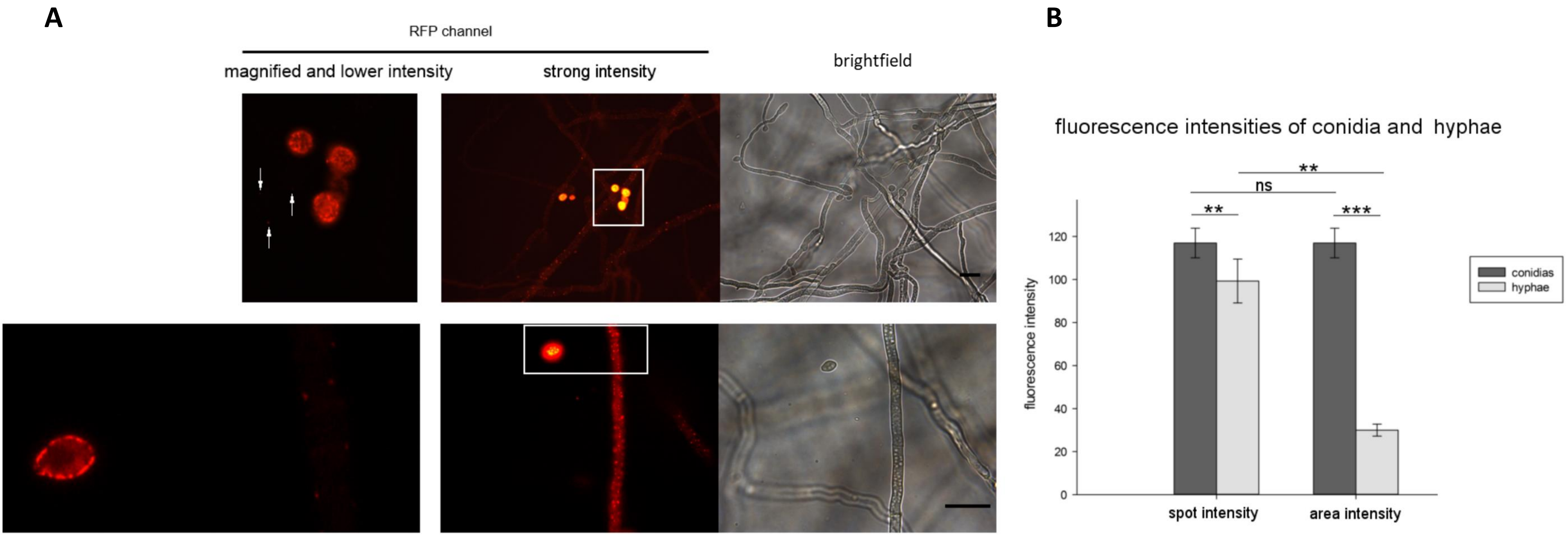

Supplementary Figure S3. Distribution of LSP-1::RFP fluorescence in conidiospores and mature hyphae.
The fluorescence in conidiospores is significant higher than that in hyphae. **A**: CLSM images of conidiospores and hyphae. Exc.: 543 nm Em.: 590-610 nm; Magnification of corresponding box regions is shown on the left side. The arrows point out some of the fluorescent spots in hyphae. **B**: Fluorescence intensity analysis of conidia and hyphae. Measurements correspond to the CLSM images in (**A**). Spot intensity is defined as the fluorescence intensity of each eisosomal spot, while area intensity is defined as the average intensity of the conidia or hyphae. Mean ±SD; n=7; t-test $**p \leq 0.01$, $***p \leq 0.001$; ns: not significant; scale bars: 20 µm

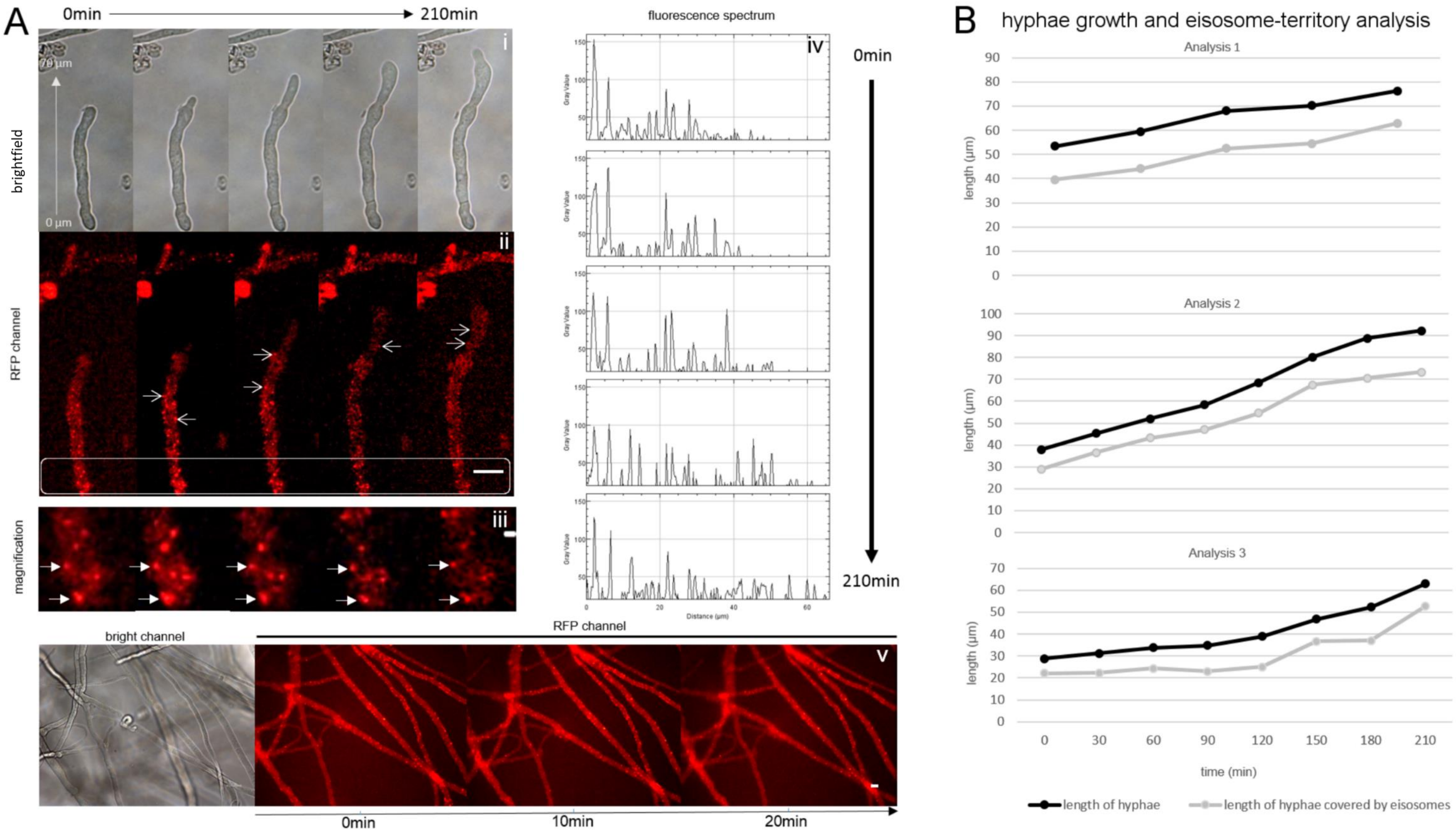

Supplementary Figure S4. Time-lapse analysis of germinating hyphae and mature hyphae.

A: CLSM images and fluorescence intensity profile analyzes of hyphae. Exc.: 543 nm Em.: 590-610 nm; **i.** Brightfield **ii.** LSP-1::RFP distribution in young hyphae within 210 min. Newly formed eisosomal spots are indicated by arrows. **iii.** Magnification of the oldest part (box region in **ii.**) of the young hyphae to different time points. The arrows point out fluorescent spots, which did not change their localisations during the time-lapse analysis. **iv**. Fluorescence intensity profile analyzes of the germinating hyphae. Left side of hyphal surface was analysed, the long arrow indicates the direction of the analysis. New fluorescent spots formed at the newly germinated part of the hyphall tube. **v.** Eisosome distribution in the mature hyphae within 20 min of investigation. Localisation of eisosomes did not change and no newly formed eisosomes could be detected. **B.** The elongation of hyphae and the formation of eisosomes during time-lapse analyzes (Analysis 1 corresponding to A-ii, captures corresponding to analyzes 2 and 3 are not shown). Scale bars: 10 µm

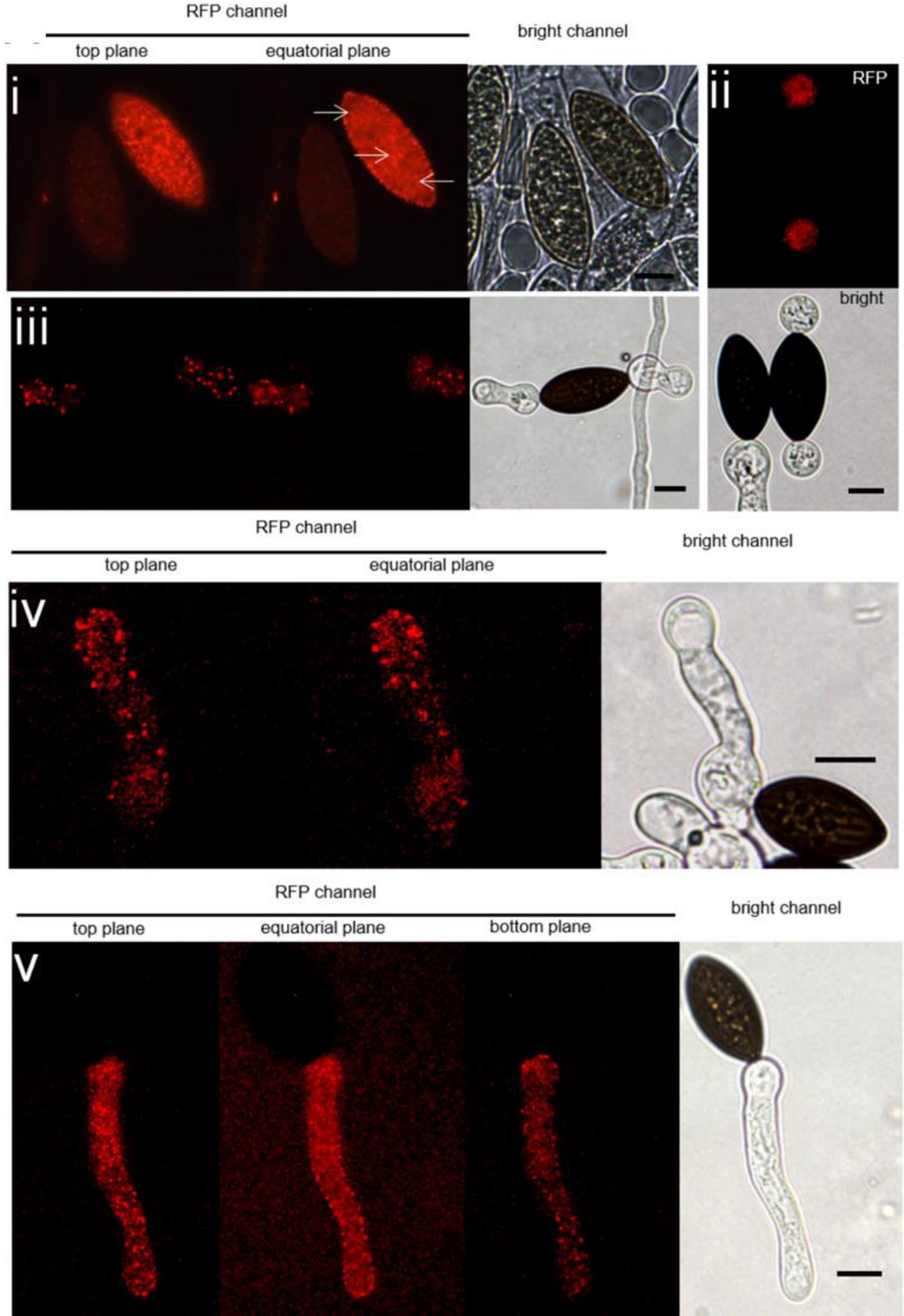

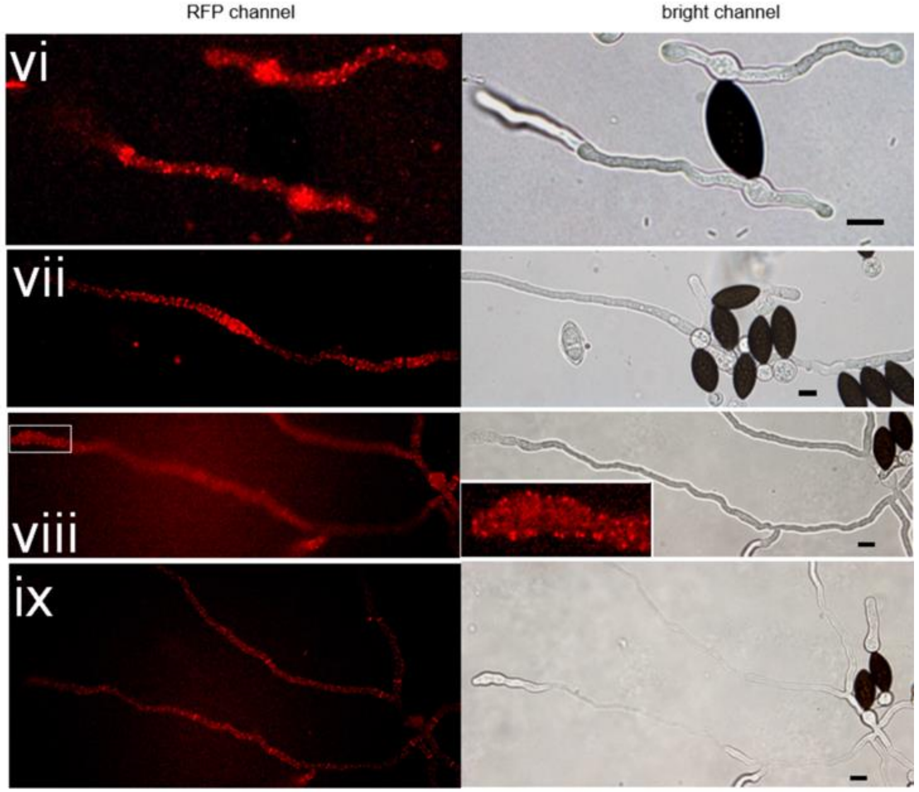

Supplementary Figure S5. Eisosome distribution in ascospores and in hyphae germinated from ascospores. CLSM images of ascospores and hyphae. Exc.: 543 nm Em.: 590-610 nm; **i.** LSP-1 is localized at the cell mambrane and in the cytoplasm of young ascospores. **ii.** The germinating buds of an ascospore. Fluorescence could not be detected in the ascospore due to melanine accumulation. **iii–ix**. Later developmental stages of germinating hyphae. **viii** and **ix** Captures of the same hyphae in different focal planes (viii focus on tip, ix focus on older hyphae part). Scale bars: 10 µm

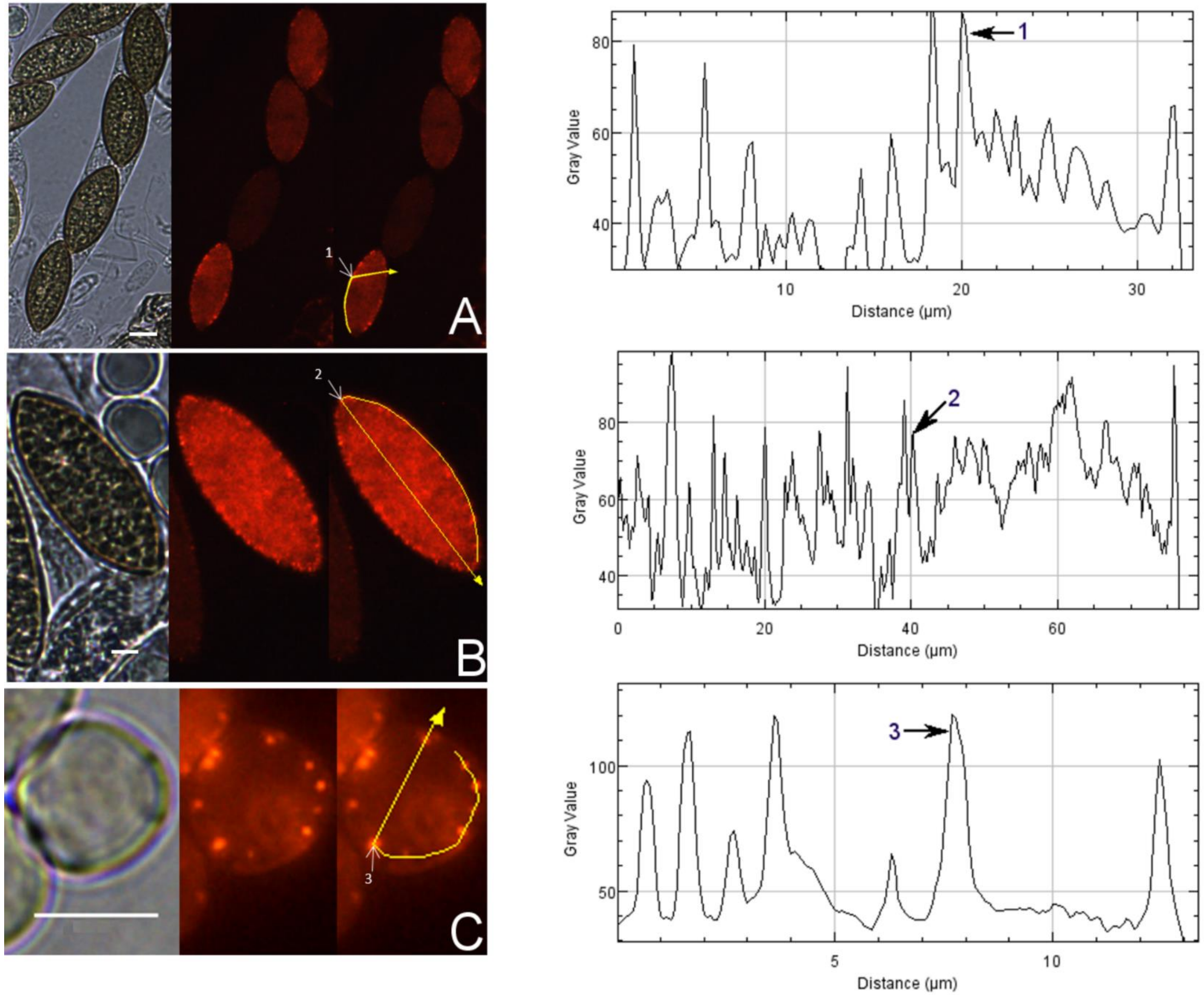

Supplementary Figure S6. Eisosome distribution in ascospores and macroconidia.
CLSM images and intensity profiles of LSP1::RFP distribution in asco- and conidiospores. Arrow lines indicate the routes and directions of intensity profile analyzes. Numbered arrows show the demarcation fluorescent spots between cell membrane and cytoplasm in both CLSM images and intensity profiles. Numbers and distance in fluorescent intensity profiles are consistent with those in the CLSM pictures. Exc.: 543 nm Em.: 590-610 nm; Scale bars: 5 μm

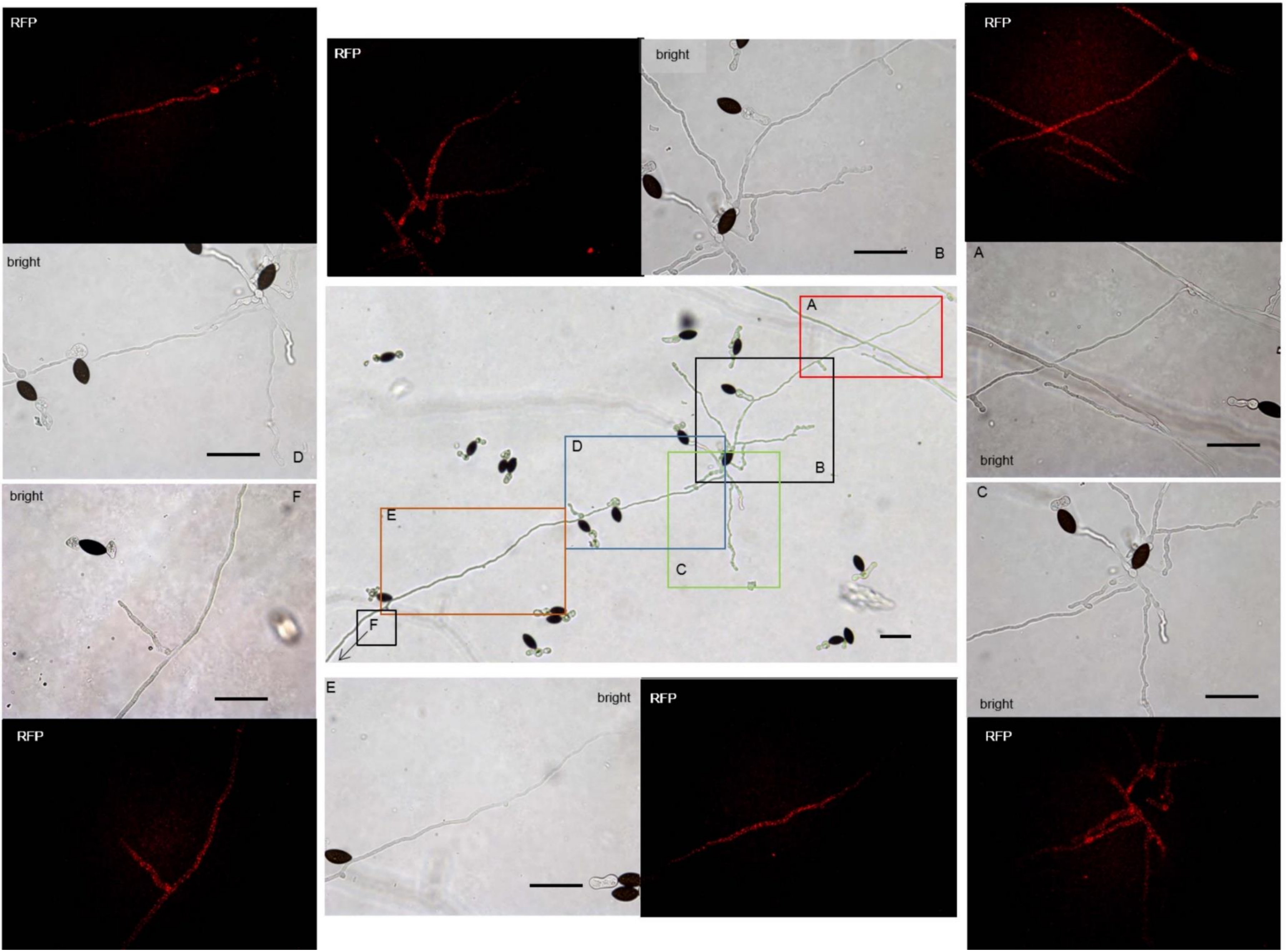

Supplementary Figure S7. Eisosome distribution in long-branched hyphae germinated from ascospores.
CLSM images of ascospores and hyphae. Exc.: 543 nm Em.: 590-610 nm; No obvious eisosome distribution differences were found at different hyphal parts. **A-E.** Magnifications are corresponding to different parts of the hyphae (box region) according to the letter of the alphabet. **F.** Magnification of the down left hyphal part, which is out of the low magnification capture. Scale bars: 50 µm

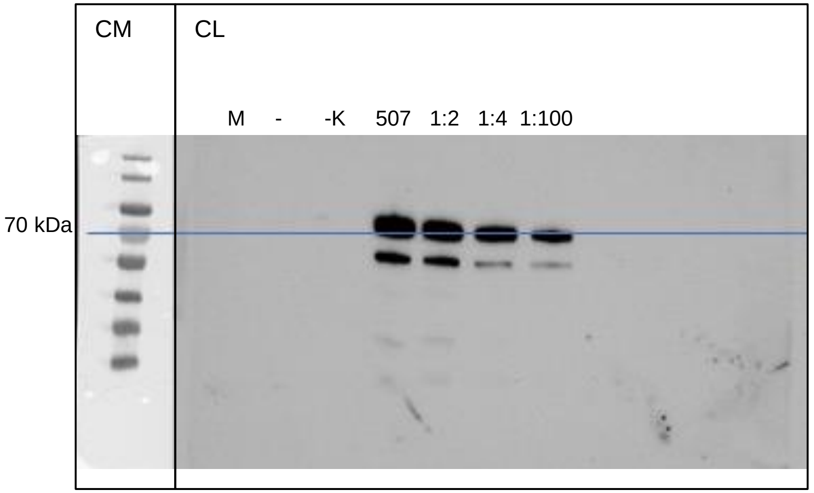

Supplementary Figure S8. Verification of full length LSP1::tRFP expression in *N. crassa*. Full Lsp1::tRFP protein size appr. 69.9 kDa. CM: colorimetric picture, only marker shown, CL: chemiluminescence M: Page Ruler (Thermo Fisher, Waltham, USA), -: empty lane, -K: negativ control (*N. crassa* FGSC #6301 total protein), 507: *N. crassa* NcT507 total protein, 1:2, 1:4, 1:100: dilution of N. crassa NcT507 total protein sample. Protein amounts in lanes –K and 1:100 are both 0.5 µg.

Supplementary Table S1. Fungal strains used in our research

Strain	Genotype	Source
FGSC6103	*his-3* (Y234M723) mat A	Fungal Genetics Stock Center
FGSC9716	*his-3* (Y234M723) mat a	Fungal Genetics Stock Center
NcT462	*ccg1 promoter:: lsp-1::rfp* mat a	This study
NcT475	*ccg1 promoter:: lsp-1::rfp* mat A	This study
NcT507	*lsp-1 promoter:: lsp-1::rfp* mat A	This study
NcT508	*lsp-1 promoter:: lsp-1::rfp* mat a	This study

Supplementary Table S2. Primers used in the study

Primer	Sequence (5‘-3‘)	Purpose
QY2942	CCTTAATTAAGGTGAGGACGGATGAATAAGAC	*N. crassa lsp-1* promoter
QY2943	TTGGCGCGCCGGTGGATTAGGTAGTATTTATCGATTG	*N. crassa lsp-1* promoter
AA2865	GGAGACAUATGCATCGAACCTACTCCATG	*N. crassa lsp-1* NCU07495
AA2866	ACAAGCCUGGCAGCAACAGGCTCAGT	*N. crassa lsp-1* NCU07495
FK2700	GACGCCGGAGCGTCTTCGGGG	pQY868 seqprimer
QY3213	CGGGATCCCGATGCATCGAACCTACTCCATGCG	*N. crassa lsp-1::rfp* fragment
QY3214	CGGAATTCCTTGTACAGCTCGTCCATGCC	*N. crassa lsp-1::rfp* fragment
QY3217	GTTCCTGAAACGCAGATGTG	pQY878 seqprimer
QY3218	CACCGCCATCATCCACAAAG	pQY868/878 seqprimer
QY3219	TCGCTCTTCACCACTAACAC	pQY868/878 seqprimer
QY3220	GGAGGTGGCGGATTATGAGG	pQY868/878 seqprimer
QY3221	GCCTCATCTACAACGTCAAG	pQY868/878 seqprimer
KK3488	GGATCCATGCACAGAACTTACTCTTT	*S. cerevisiae pil1*
KK3489	AGATCTAGCTGTTGTTTGTTGGG	*S. cerevisiae pil1*
KK2603	GAAGTGGTGGTTGTTCACGG	pKK904 seqprimer
